# Fabrication of Au/Fe_3_O_4_/RGO based aptasensor for measurement of miRNA‐128, a biomarker for acute lymphoblastic leukemia (ALL)

**DOI:** 10.1002/elsc.202100170

**Published:** 2022-05-17

**Authors:** Homayoon Soleimani Dinani, Mehrab Pourmadadi, Fatemeh Yazdian, Hamid Rashedi, Seyed Ali Seyed Ebrahimi, Javad Shabani Shayeh, Mehdi Ghorbani

**Affiliations:** ^1^ School of Chemical Engineering College of Engineering University of Tehran Tehran Iran; ^2^ Department of Life Science Engineering Faculty of New Science and Technologies University of Tehran Tehran Iran; ^3^ School of Metallurgy and Materials Engineering College of Engineering University of Tehran Tehran Iran; ^4^ Protein Research Center Shahid Beheshti University Tehran Iran; ^5^ Department of Chemical Engineering Marvdasht Branch Islamic Azad University Marvdasht Iran

**Keywords:** acute lymphoblastic leukemia, aptamer probe, electrochemical biosensor, miRNA detection, RGO nanocomposite

## Abstract

Due to their high sensitivity, simplicity, portability, self‐contained, and low cost, the development of electrochemical biosensors is a beneficial way to diagnose and anticipate many types of cancers. An electrochemical nanocomposite‐based aptasensor is fabricated for the determination of miRNA‐128 concentration as the acute lymphoblastic leukemia (ALL) biomarker for the first time. The aptamer chains were immobilized on the surface of the glassy carbon electrode (GCE) through gold nanoparticles/magnetite/reduced graphene oxide (AuNPs/Fe_3_O_4_/RGO). Fast Fourier transform infrared (FTIR), X‐ray diffraction (XRD), vibrating sample magnetometer (VSM), and transmission electron microscopy (TEM) were used to characterize synthesized nanomaterials. Cyclic voltammetry (CV), square wave voltammetry (SWV), and electrochemical impedance spectroscopy (EIS) were used to characterize the modified GCE in both label‐free and labeled methods. The results indicate that the modified working electrode has high selectivity and for miRNA‐128 over other biomolecules. The hexacyanoferrate redox system typically operated at around 0.3 V (vs. Ag/AgCl), and the methylene blue redox system ran at about 0 V, were used as an electrochemical probe. The detection limit and linear detection range for hexacyanoferrate and methylene blue are 0.05346 fM, 0.1–0.9 fM, and 0.005483 fM, 0.01–0.09 fM, respectively. The stability and diffusion control analyses were performed as well. In both label‐free and labeled methods, the modified electron showed high selectivity for miRNA‐128. The use of methylene blue as a safer redox mediator caused miRNA‐128 to be detected with greater accuracy at low potentials in PBS media. The findings also show the substantial improvement in detection limit and linearity by using reduced graphene oxide‐magnetite‐gold nanoparticles that can be verified by comparing with previous studies on the detection of other miRNAs.

**Abbreviations**
ALL: acute lymphoblastic leukemia
AML: acute myeloid leukemia
Apt/AuNPs/Fe_3_O4/RGO: aptamer/gold nanoparticles/magnetite/reduced graphene oxide
BSA: bovine serum albumin
CML: chronic myeloid leukemia
CLL: chronic lymphoblastic leukemia
CV: cyclic voltammetry
EIS: electrochemical impedance spectroscopy
FTIR: fast fourier transform infrared
GCE: glassy carbon electrode
GO: graphene oxide
LOD: limit of detection
LPS: lipopolysaccharide
PBS: phosphate buffered saline
PSA: prostate‐specific antigen
SELEX: systematic evolution of ligands by exponential enrichment
SWV: square wave voltammetry
XRD: X‐ray diffraction
VSM: vibrating sample magnetometer
2D: two dimensions

## 1 INTRODUCTION

As shown by the fact that 8.2 million out of 14 million new cancer cases each year do not survive, cancer is one of the major causes of death globally [1]. Leukemias are cancers that develop in the bone marrow and are categorized as hematological malignant clonal diseases. In this malady precursor, lymphoblasts, blocked at the beginning of the differentiation process, subsequently proliferate quickly and supersede normal hematopoietic cells of the bone marrow. Acute myeloid leukemia (AML), chronic myeloid leukemia (CML), acute lymphoblastic leukemia (ALL), and chronic lymphoblastic leukemia (CLL) are the four types of leukemia that are traditionally categorized based on their morphologic and genomic characteristics (CLL) [2, 3]. ALL is one of the most common types of leukemia, which is the main malignant disease arising among children. In every 2000 children under the age of 15, one is suffering from leukemia. ALL in adults, on the other hand, is nearly one‐fourth of children and accounts for <20% of whole cases of leukemia in adults. For ALL, the peak of incidence is between 1 and 4 years old for children and over 50 years old for adults [4, 5]. The rate of survival, especially for children and adolescents, has been drastically increased in the past six decades (from a median survival of 2 months from diagnosis up to 90% long‐term overall survival for childhood ALL and a cure rate of 20%–40% for adults) [6, 7, 8].

A crucial point in cancer treatment methods is early diagnosis, especially before cells can split off from malignant tumors and metastasize all over the patient's body [9]. Metastasis occurring by the spread of tumor cells from the original site and subsequently emerging and developing new colonies in secondary tissues. In general, 90% of deaths caused by cancer occur after the metastatic process because of the complexity of tumor development. More than 200 types of cancer have been identified that vary in cell origin, mutations, and genetic variability, as well as the crosstalk with the tumor microenvironment, making current treatments inefficient [10]. However, conventional methods do not satisfy the criteria of early detection. Most of these conventional methods, such as morphological analysis of the cells under an optical microscope [11], DNA sequencing [12], flow cytometry [13], fluorescence in situ hybridization [14], polymerase chain reactions [15], microarrays of antibodies [16] and immunohistochemistry [17] are expensive, laborious and time‐consuming. Other demerits of conventional methods we should take into account are the requirement for advanced instrumentation and multi‐step processing. Therefore, it is essential for any research in the field of diagnostics to develop a cost‐effective, simple, and rapid procedure by high selectivity and sensitivity [18], as current research illustrates the possibility of achieving these requirements in ALL diagnosis.

A promising way for the diagnosis and prognosis of many types of cancer is developing electrochemical biosensors [19, 20] due to their high sensitivity, simplicity, portability, self‐contained, and low‐cost [21, 22, 23]. Analogous to other biosensors, electrochemical biosensors comprise of two indispensable components, the biological recognition element (the bio‐receptor) and a transducer (the electrode) connected to an electronic reader device [24]. The reaction between bio‐receptor and analyte (biomarker) on the surface of the electrode generates a biological signal, which is converted to an electronic signal through the transducer. This signal is subsequently amplified to yield high sensitivity and specificity [25, 26].

Generally, miRNAs are short single‐stranded endogenous non‐coding RNAs that contain 18–25 nucleotides. miRNAs are most familiar because of their post‐transitional role in negatively regulating gene expression [27, 28, 29]. A bead‐based cytometric for comparison between mRNA and miRNA expression profiles in different types of cancer illustrated that miRNAs are a far better choice for diagnosis purposes because they can classify cancers more accurately and reliably make a correlation with the stage of cancer. The prominent miRNAs that can be used for leukemia diagnosis are miRNA‐128a, miRNA‐128b, miRNA‐223, let‐7b, miRNA‐155, and miRNA‐24. According to genome‐wide miRNA expression analysis, using the bead‐based flow cytometric method, miRNA‐223, miRNA‐128a, and miRNA‐128b are the best biomarkers for the segregation of distinct mechanisms of leukemogenesis in ALL and AML [30, 31, 32]. miRNA‐128 has a vital role in nervous system development, while its expression change in tumor cells via several genetic and epigenetic activities. Through cell proliferation, differentiation, metabolism, and apoptosis, altered miRNA‐128 has a significant impact on oncogenesis. It is proven that in ALL, the expression level profile of miRNA‐128a and miRNA‐128b (letters a and b are referred to different genes that are encoded by) notably increase, so it can be used as a biomarker for ALL detection and progress [33, 34, 35]. For the complementary substance, an aptamer with a particular sequence of nucleotides has been chosen for the bio‐receptor part, which has a high binding affinity and specificity to the miRNA‐128 [36]. Aptamers are the single‐stranded sequence of 20–80 nucleotides. Aptamers artificially are selected and produced via the Systematic evolution of ligands by exponential enrichment (SELEX), which is an in vitro molecular method [37]. The most significant advantages of aptamers comparing to other bio‐receptors such as antibodies are nontoxicity, high stability in harsh conditions, a wide range of targets, cost‐effective, small size, the capability of modification with various tags, low immunogenicity, and long half‐life [38, 39, 40, 41].

In recent years nanomaterials and nanocomposites attracted considerable attention in the fabrication of biosensors with higher sensitivity and specificity due to their exceptional properties [42, 43, 44]. Compared with conventional sensors, nanomaterial‐modified electrochemical biosensors offer benefits including high conductivity, stability, and biocompatibility because of their superior area‐to‐volume ratio results in higher catalytic performance and sensing response as well as improved optical, magnetic, and electrical properties [45, 46]. Properties including particular chemical composition, surface texture, crystal structure perfection, crystallographic axis orientation make nanocomposites an excellent choice for effective surface modification of electrochemical biosensor electrodes [46].

**PRACTICAL APPLICATION**

- Studying the effects of modifying the working electrode with RGO, AuNPs, and Fe_3_O_4_ NPs separately.
- Synthesis of Au/Fe_3_O_4_/RGO with a facile and novel protocol.
- Quantitative determination of miRNA‐128 concentration for the first time.
- Using an exclusive aptamer probe as a bioreceptor molecule for precise recognition.
- Reaching a fast and reliable acute lymphoblastic leukemia diagnosis method.
- High repeatability and low LOD make this method proper for commercialization.

Graphene is a hexagonal carbon network that looks like a honeycomb structure, which is indeterminately extended in two dimensions (2D). The physical and chemical properties of graphene and its derivatives (such as reduced graphene oxide [RGO]) include high sensitivity, remarkable selectivity and stability, low overpotential, wide potential window, minimal capacitive current, and outstanding electrocatalytic activity [47, 48]. It offers various other fascinating properties such as considerable specific surface area, excellent electrical conductivity and transparency, superior mechanical strength and durability, strong ambipolar electrical field effect, good thermal conductivity, and outstanding electronic properties [49, 50]. The hybridization of graphene and metal nanoparticles leads to the nanocomposite with excellent surface area and improved the kinesis of charge carriers as well as firm electron transfer kinetics [46, 51, 52].

Magnetite (Fe_3_O_4_) nanoparticles are known magnetic materials that have been used increasingly in the biosensor field mainly because of their high surface area, high biocompatibility, low toxicity, easy preparation, and high adsorption ability [53]. Also, accessibility to active surface area and electron transfer rate between the electrode surface and redox reaction can be dramatically enhanced by Fe_3_O_4_ nanoparticles on that interface [54, 55, 56]. Metal oxide nanomaterials usually have higher catalytic activity than single‐component nanomaterials (like gold nanoparticles), which is very useful for homogeneous dispersion of noble metal nanoparticles on a specific surface [57, 58]. The synergetic effect occurs at the border of metal and oxide support which results in high catalytic activity [46, 59].

On the other hand, possessing properties like large specific area, outstanding biocompatibility, high surface free energy, complete recovery in biochemical redox reactions, excellent chemical stability, and high capabilities in making bonds with multiple functional groups make gold nanoparticles (AuNPs) one of the best candidates in developing electrochemical biosensors [38, 60, 61, 62]. Gold nanoparticles and their composites also are very suitable for miniaturized sensing platforms, especially electrochemical biosensors, because of their wide electrochemical potential range, simple preparation, easy fabrication process, and high catalytic activity [63, 64, 65, 66]. In nanohybrids, all advantages of the comprising species can be magnified due to the synergic interaction between them [67]. For instance, nanohybrids can be very efficient in immobilizing the bioreceptors and adsorbing the desired biomolecules at the same time, which makes the fabricated biosensor very selective [68, 69].

**FIGURE 1:**
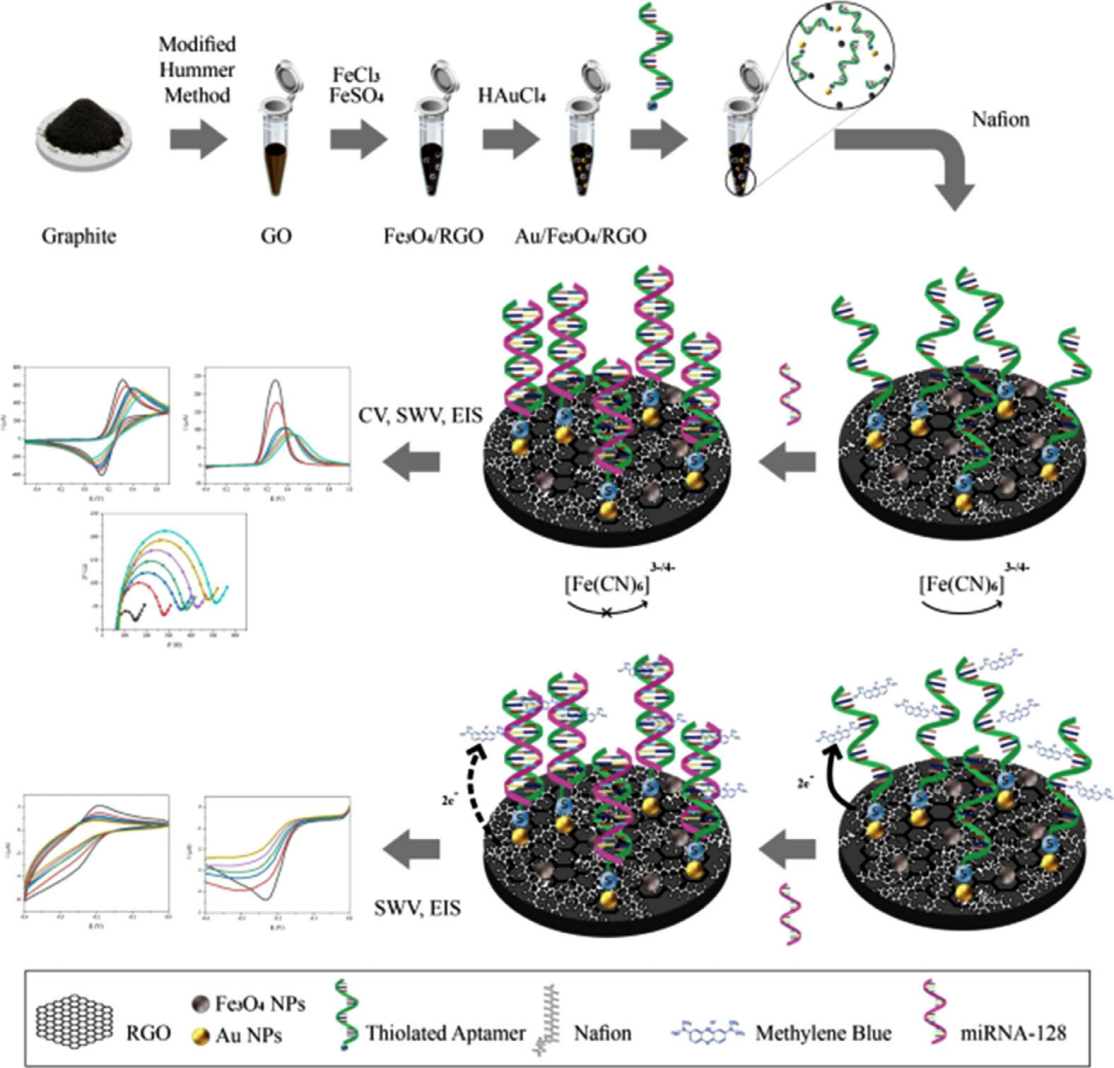
Graphical abstract of electrode surface modifications and electrochemical analysis, including the synthesis procedure of gold/magnetite/reduced graphene oxide nanocomposite and two electrochemical methods (label‐free and labeled) for determination of miRNA‐128 concentration

One of the essential parts of this research is the state‐of‐the‐art application of Au/Fe_3_O_4_/RGO nanocomposites with a synthesis protocol, which enhanced the biosensor performance significantly. The fundamental principle of the present research is to determine the ALL biomarker (miRNA‐128) concentration in two electrolytes (potassium ferrocyanide and phosphate‐buffered saline, abbreviated as PBS) with different approaches (label‐free and labeled). Quantitative determination of this biomarker concentration is accomplished for the first time, and it can be beneficial for the diagnosis and prognosis of ALL as well as distinguish it from other types of cancers, especially AML [70, 71]. Electrode surface modifications and electrochemical analysis, including the synthesis procedure of gold/magnetite/reduced graphene oxide nanocomposite and two electrochemical methods (label‐free and labeled) for determination of miRNA‐128 concentration can be seen in Figure 1.

## 2 MATERIALS AND METHODS

### 2.1 Reagents and solutions

Analytical grade graphite powder, sodium borohydride (NaBH_4_), potassium chloride (KCl) (as the preservative solution for the reference electrode), sulfuric acid (H_2_SO_4_), hydrogen peroxide (H_2_O_2_), hydrochloric acid (HCl), chloroauric acid (HAuCl_4_.4H_2_O), potassium hexacyanoferrate (II) (K_4_[Fe(CN)_6_]), Nafion (C_7_HF_13_O_5_S•C_2_F_4_), glucose, bovine serum albumin (BSA), prostate‐specific antigen (PSA), potassium permanganate (KMnO_4_), ethanol (C_2_H_5_OH), iron (III) chloride (FeCl_3_), iron(II) sulfate (FeSO_4_), aluminum oxide (Al_2_O_3_), sodium hydroxide (NaOH), phosphorus pentoxide (P_4_O_10_), potassium persulfate (K_2_S_2_O_8_), were all purchased from Sigma‐Aldrich (Germany http://www.sigmaaldrich.com), and were used as received. All solutions were prepared using deionized water. The miRNA‐128 complementary aptamer with a thiol modifier (C_6_SH) and the lipopolysaccharide (LPS) aptamer (dissociation constant or Kd = 12 nM) with 6'NH_2_ modification both with HPLC purification was synthesized by bio basic inc. (Canada, https://www.biobasic.com) with following sequence respectively: (5'AAAGAGACCGGTTCACTGTGA‐(CH_2_)_6_‐SH3') and (CTTCTGCCCGCCTCCTTCCTAGCCGGATCGCGCTGGCCAGATGATATAAAGGGTCGCCCCCCAGGAGACGAGATAGGCGGACACTOD5 HPLC 5' Mod Amino Modifier ‐ NH_2_C_6_). miRNA‐128 with the sequence of UCACAGUGAACCGGUCUCUUU [72] were ordered from Microsynth Co. (Balgach, Switzerland, https://www.microsynth.ch). The lyophilized aptamer and miRNA‐128 powders were diluted in by phosphate buffered saline (PBS), (NaCl 0.138 M; KCl 0.0027 M); pH = 7.4 and stored at −20°C. The deionized water used in each step was obtained from a deionizer machine produced by Chemia rahavard Co. (Tehran, Iran, http://www.crp.ir).

### 2.2 Apparatus

A glassy carbon working electrode (diameter = 2 mm) was ordered from Detect Co. (Tehran, Iran, http://www.detectco.com). A Ag/AgCl electrode (Ionode Co., version IJ‐14, Australia, https://ionode.com) was used as the reference electrode, and a platinum electrode version IRI.2000‐E was used as the counter electrode. An Ivium (vertex one) electrochemical potentiostat (Eindhoven, Netherlands, https://www.ivium.com) coupled with Ivium software used for electrochemical measurements, including cyclic voltammetry (CV), square wave voltammetry (SWV), and electrochemical impedance spectroscopy (EIS). The aptamer and miRNA‐128 were aliqutated under the laboratory fume hood (RAAD TEB NOVIN Co., Iran, http://www.raadlabco.com) in a sterile condition. A centrifuge device manufactured by Clement Co. (GS200 class) (Australia, https://www.clements.net.au) was used in separating nanosized particles. A Cana way pH meter acquired from SAT Co. (Iran, http://www.sat.co.ir) was used. An ultrasonic bath class Eurosonic 4D manufactured by Euronda Co. (Italy, https://prosystem.euronda.com) was used for homogenizing the nanoparticle solutions. EIS experiments were conducted using an electrochemical cell connected to potentiostat in the 100 kHz ‐ 15 mHz frequency range with a 5 mV perturbation amplitude at the oxidation potential of the oxidation peak current of CV curves.

### 2.3 Synthesis of Au/Fe_3_O_4_/RGO nanocomposite

**FIGURE 2:**
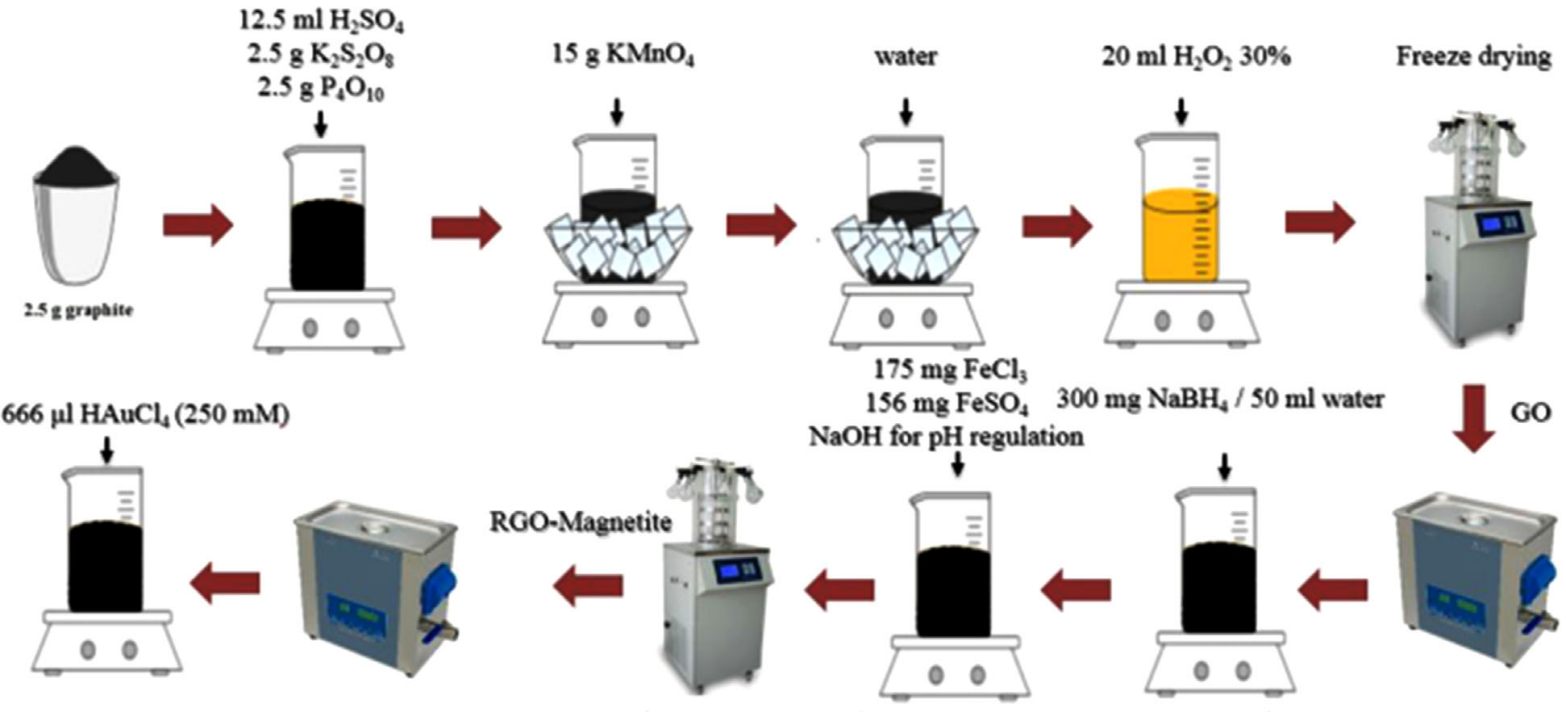
Schematic procedure of Au/Fe_3_O_4_/RGO synthesis

Graphene oxide (GO) was synthesized by the implementation of Hummer's method [73]. In our experiment, 50 mg GO was added in 150 mL of deionized water, and after 2 h of ultrasonication, a homogeneous dispersion was obtained [74]. This was followed by the dropwise addition of 50 mL of 0.1586 mol L^–1^ of aqueous NaBH_4_ solution. The mixture stirred again for 1 h at 80°C to make sure GO reduced entirely. Next, a mixture of 175 mg FeCl_3_.6H_2_O and 156 mg FeSO_4_.7H_2_O was dissolved in 50 mL of deionized water. After a homogeneous solution was obtained, the pH was adjusted to 10.55 by adding NaOH dropwise. The solution was then heated at 80°C for 2 h. After centrifuging and rinsing one time with ethanol and then several times with deionized water, the obtained reduced graphene oxide/Fe_3_O_4_ dried in the freezer. Deionized water was added to 117 mg of Fe_3_O_4_/RGO to reach the volume of 27 mL. The mixture was ultrasonicated for 10 min, following by the dropwise addition of 666 μL of 250 mM HAuCl_4_.6H_2_O under constant stirring. The stirring continued for 1 h. The produced Au/Fe_3_O_4_/RGO separated and washed by several centrifuging and rinsing by ethanol and deionized water, finally dried in the freeze dryer. All sample tubes, microtubes, and glassware were autoclaved to mitigate the impact of RNases on the stability of miRNAs. Figure 2 shows the procedure of Au/Fe_3_O_4_/RGO synthesis.

### 2.4 Characterization and electrochemical measurements

Fourier‐transform infrared (FTIR) spectroscopy was recorded using a spectrometer at room temperature with a resolution of 1 cm^–1^ (Thermo Incol, USA, https://www.thermofisher.com). X‐ray diffraction (XRD) analysis was performed on an X‐ray diffractometer operating at 40 kV and 30 mA, using a Cu Kα radiation and a Ni filter (STOE, Germany, https://www.stoe.com). The magnetic features of Fe_3_O_4_/RGO and Au/Fe_3_O_4_/RGO nanocomposites were tested with a vibrating sample magnetometer (VSM) at 298 K. Transmission electron microscopy (TEM) was used to reveal the surface morphology, composition, and internal structure of the nanocomposites (TEM, Philips Tecnai G220, operated at 120 kV, https://www.jeolusa.com).

The electrochemical detection techniques that had been utilized were CV, SWV, and EIS. These techniques were applied to a three‐electrode device consisting of a Ag/AgCl reference electrode, a glassy carbon working electrode (2 mm geometric diameter), and a platinum counter (auxiliary) electrode. All electrochemical experiments were performed at room temperature (20 ± 2°C).

### 2.5 Incubation and preparing the aptamer/Au/Fe_3_O_4_/RGO

In our work, 2 μL of 100 μM aptamer (apt) solution was added to 98 μL of 2 mg.mL^–1^ Au/Fe_3_O_4_/RGO, and the resulting solution was placed in a refrigerator at 4°C. The solution was incubated to promote spontaneous bonding between the thiolated 3' end of the aptamer sequence with the gold nanoparticle molecules.

### 2.6 Electrode modification procedure

**FIGURE 3:**
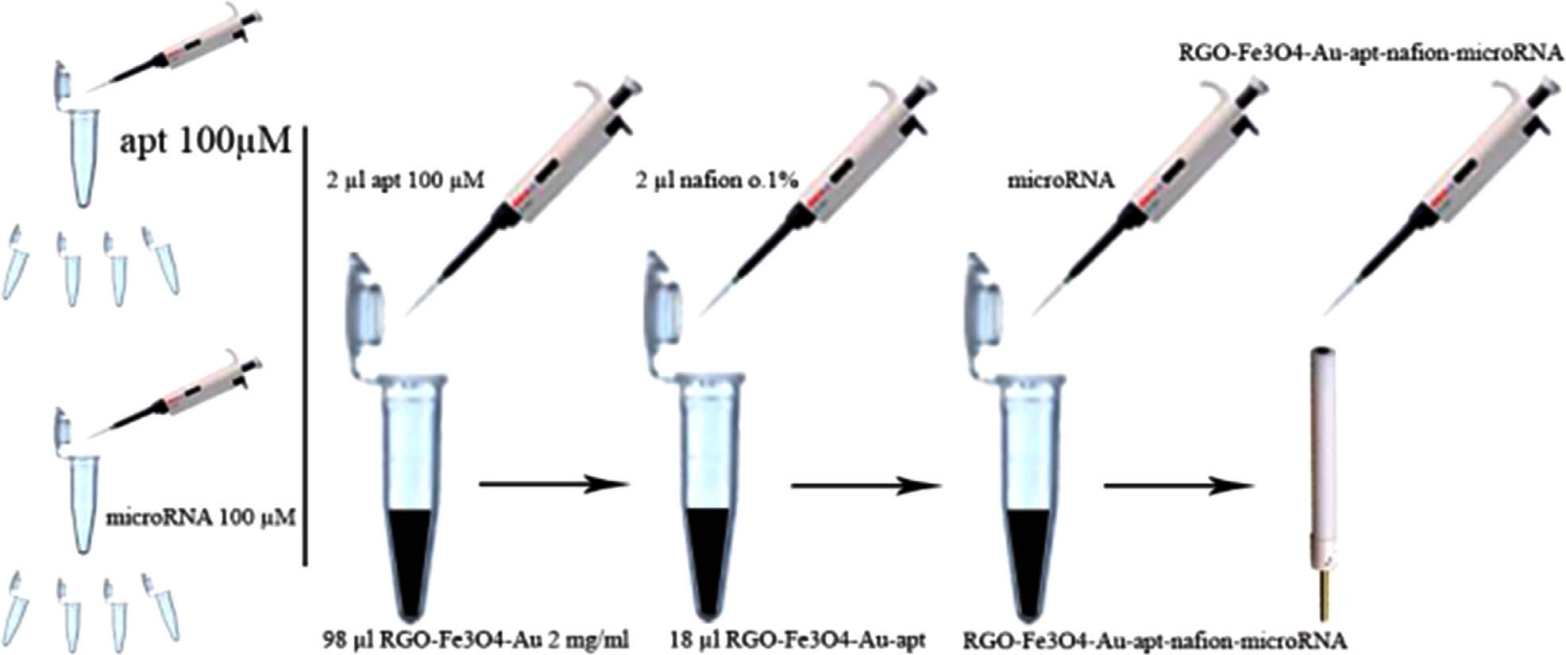
Schematic procedure of electrode modification

For further modification of electrode, 2 μL of 0.1%wt. Nafion (1:10) was added to 18 μL of Apt/Au/Fe_3_O_4_/RGO solution, and 15 min was allowed to maximize the effect on the nanocomposite, and after that, this volume was applied to the glassy carbon electrode surface, which was allowed to dry at room temperature. In all electrochemical analyses, 4 μL of miRNA‐128 sample was delivered to the surface of the modified electrode, and after optimum hybridization time (30 min), the electrode rinsed in PBS (pH = 7.4) to release unbonded materials at the surface of the electrode. The modified electrode was eventually ready for electrochemical analyses. Procedure of electrode modification can be seen in Figure 3.

### 2.7 Response time

In the first step, 4 μL of the solution containing the analyte at constant concentration was dropped onto the working electrode and allowed to incubate for 5 min, and the SWV test in the potential range of ‐0.4 to 1 V was taken. This procedure was repeated for 15, 25, 35, 45, and 55 min, and the maximum difference in the electron current when the analyte was on the surface was compared to that of the nanoparticles alone at different times of calculation.

### 2.8 Concentration analysis by label‐free method

CV, SWV, and EIS tests were performed in potassium ferricyanide electrolyte to obtain the base signal on the working electrode prior to the surface modification of the polished surface. First, 4 μL of the Apt/Nafion/Au/Fe_3_O_4_/RGO solution was applied to the center of the working electrode. After being dried under a lamp, CV, SWV, and EIS measurements were performed. In the next step, 10 μL of 0.1 fM miRNA‐128 was applied to the center of the electrode, and after 30 min of optimum incubation under a cap, CV, SWV, and EIS tests were again performed. This procedure repeated for 0.3 fM, 0.5 fM, 0.7 fM, and 0.9 fM, respectively.

### 2.9 Concentration analysis by labeled method

Due to the disadvantages of the ferrocyanide/ferricyanide redox couple, such as high toxicity, impracticality of the label, etc., it is better to eliminate these disadvantages by using an alternative redox marker. One of the best alternatives for this goal is methylene blue. The toxicity of this material is very low, has the potential to be labeled, and has prominent optical properties. For this purpose, after the electrode modification as described in the previous section, the electrode was placed in a beaker containing 2.5 mM methylene blue solution for 45 min. After removing the excess methylene blue, the electrode was incubated in phosphate buffer (pH 4.7, 100 mM) for 5 min. Next, the base signal of CV and SWV has been obtained, and these two tests were conducted using 0.01 fM, 0.03 fM, 0.05 fM, 0.07 fM, and 0.09 fM of miRNA‐128, respectively.

## 3 RESULTS AND DISCUSSION

**FIGURE 4:**
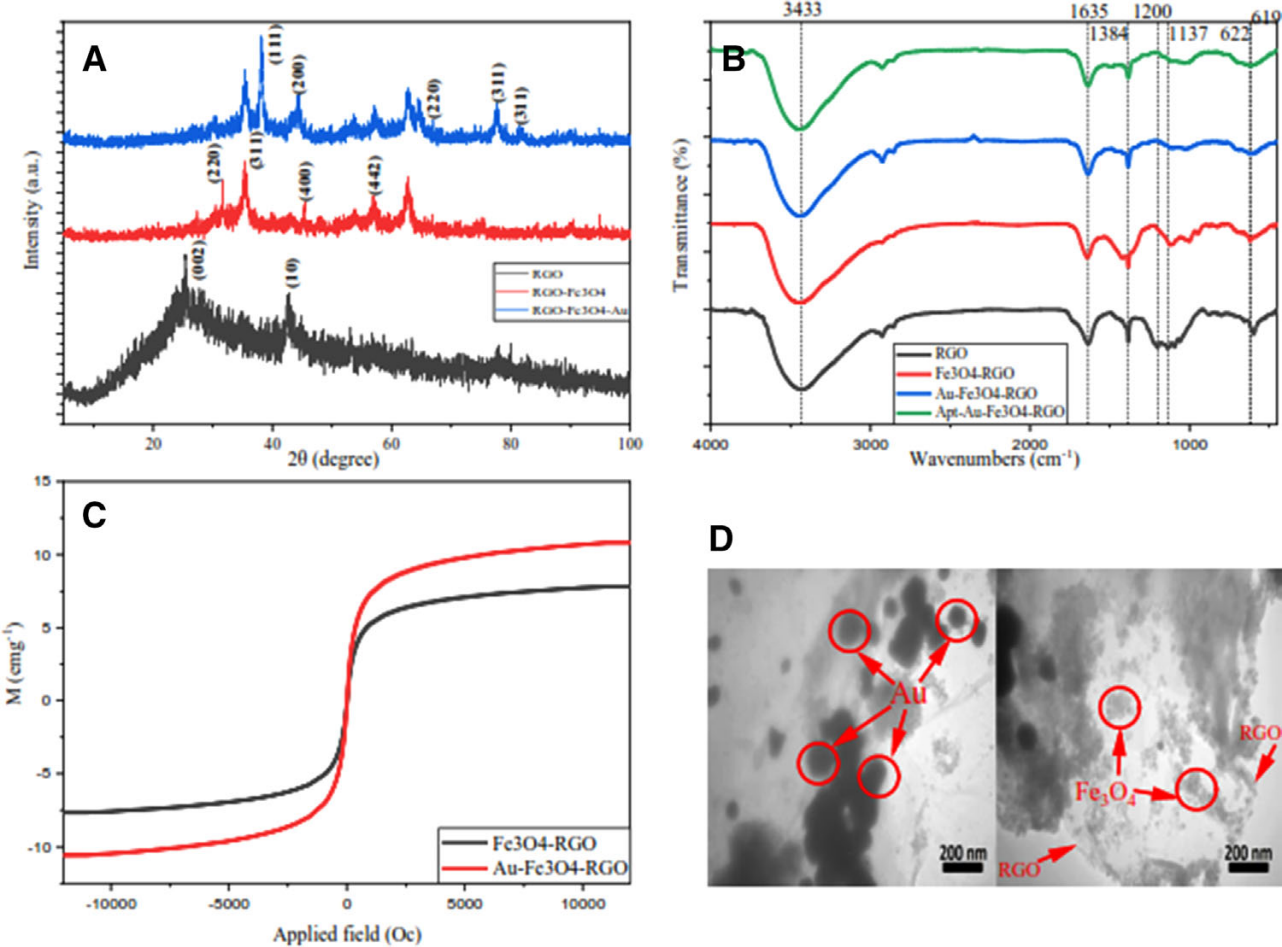
(A) XRD patterns of RGO, Fe_3_O_4_/RGO and Au/Fe_3_O_4_/RGO NPs, (B) FTIR spectra of RGO, Fe_3_O_4_/RGO, Au/Fe_3_O_4_/RGO, and Apt/Au/Fe_3_O_4_/RGO NPs, (C) VSM graphs of Fe_3_O_4_/RGO and Au/Fe_3_O_4_/RGO NPs, (D) and TEM graphs of Au/Fe_3_O_4_/RGO NPs

For obtaining comprehensive information about the chemical composition and crystalline structure of synthesized nanomaterials, XRD analysis was performed (Figure 4A). The XRD spectra show (002) diffraction peak at 2θ = 25.4230°, indicating the distance between RGO layers, and (10) diffraction peak at 2θ = 42.6026°, indicating a short‐range order in stacked RGO layers. The diffraction patterns were processed using xpert highscore plus software [75]. Decoration of RGO sheets with Au and Fe_3_O_4_ nanoparticles caused to appearance of new reflection analysis in Fe_3_O_4_/RGO and Au/Fe_3_O_4_/RGO XRD patterns. In the plot related to Fe_3_O_4_/RGO, peaks are observed in the 2θ = 31.6747°, 2θ = 35.4403°, 2θ = 45.4943°, 2θ = 56.9773°, and 2θ = 62.8807°, indicating (011), (113), (402), (512), and (022) sheets [76]. As can be seen, the pattern of Fe_3_O_4_/RGO shows apparent diffraction peaks of Fe_3_O_4_, and the peak locations and relative intensities follow the standard XRD magnetite data (COD database code: 1526955). In the graph related to reduced Au/Fe_3_O_4_/RGO, several peaks are observed in 2θ = 38.1491°, 2θ = 44.4476°, 2θ = 64.6450°, 2θ = 77.6303° and 2θ = 81.6198°, which represent the crystal sheets (111), (020), (220), (131) and (222) of AuNPs. So, the AuNPs are well placed on the RGO surface (COD database code: 9008463) [77].

In order to confirm the nanocomposite synthesis, FTIR spectroscopy was performed at each step of the synthesis process to find out the functional groups present in the material as well as the interactions between them. As can be seen in Figure 4B (black curve), there is an absorption band in 3433 (cm^–1^) confirming the stretching vibration between the hydrogen and oxygen atoms in the hydroxyl (OH) functional group, which is a result of reactions with water molecules have occurred. Also, based on 1137 (cm^–1^) and 1200 (cm^–1^) peaks were related to the stretching vibration in the C‐O bond in the epoxy and alkoxy functional groups. In addition, the peak at 1635 (cm^–1^) is related to the stretching vibration at the C═O bond in the carbonyl and carboxyl functional groups. The C═O bond facilitates the stabilization of gold nanoparticles and biological molecules such as aptamers by covalent or electrostatic bonding on the surface of the nanocomposite [78, 79] and has therefore been reduced in blue and green curves. The peak shown in 1384 (cm^–1^) is also related to the aromatic C‐OH bond. The electronic structure of carbon atoms alters from sp^3^ to sp^2^ due to the reduction of GO sheets and C═O groups, and therefore the magnitude of C═C bonds increased that appeared at the Fe_3_O_4_/RGO spectrum [80]. The peak at 619 (cm^–1^) in the Fe_3_O_4_/RGO curve (red) illustrates the presence of Fe‐O bonding, which confirms the bonding of Fe_3_O_4_ on the RGO sheets [81]. Based on the Au/Fe_3_O_4_/RGO curve, it is apparent that the peaks corresponding to the C═O bond have decreased in comparison with the black curve, and also a peak at 622 (cm^–1^) wavelength corresponding to the Au‐O‐Au bond confirm that gold nanoparticles are stabilized adequately on the surface of the nanocomposite [82]. In the Apt/Au/Fe_3_O_4_/RGO curve (green), the peaks corresponding to the C═O bond were further weakened, confirming the binding of aptamer molecules.

VSM analysis was used to investigate the magnetic properties of Fe_3_O_4_/RGO and Au/Fe_3_O_4_/RGO nanocomposites, the results of which can be seen in Figure 4C. Gold has diamagnetic properties, and it becomes ferromagnetic after conversion to nano‐size [83]. As a result, by bonding gold nanoparticles onto the surface of the reduced Fe_3_O_4_/RGO sheets, the magnetic properties of the nanocomposite improves, and this can facilitate the electron transfer through the induced current. According to the VSM curves (Figure 4C), the values ​​of Ms (Magnetization) and Mr (Retentivity) for Fe_3_O_4_ are 7.7981 and ‐0.0688, and for Au/Fe_3_O_4_/RGO were 10.8112 and ‐0.0553, respectively. These values ​​clearly confirm the increased magnetic properties of the nanocomposite due to the addition of gold nanoparticles.

The low and high magnification transmission electron micrographs of Au/Fe_3_O_4_/RGO are shown in Figure 4D. As can be seen, the two‐dimensional RGO sheets are well separated and decorated by a large number of spherical Fe_3_O_4_ and Au nanostructures. The uniform distribution of the Fe_3_O_4_ nanoparticles with an average size of 20 nm is shown in these images (this result is consistent with the crystallite size calculated from Scherer's equation for Fe_3_O_4_/RGO nanocomposite). It is evident that instead of merely blending or mixing, the Fe_3_O_4_ nanoparticles are properly entrapped inside the RGO sheets. The RGO sheets act as a conductive channel and firmly anchor the Fe_3_O_4_ nanoparticles. The results show 100 nm gold nanoparticles homogeneously distributed on the RGO sheets. Modification of RGO sheets by AuNPs gave rise to the RGO sheets' conductivity, and in addition, AuNPs provide a suitable platform for aptamer decoration.

### 3.1 Electrochemical characterization of modified electrode

**FIGURE 5:**
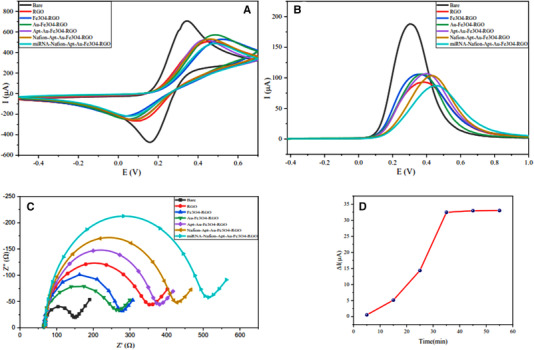
The electrochemical characterization of the nanocomposite. (A) CV analysis at the scan rate of 50 mV.s^−1^. (B) SWVanalysis at each step of electrode modification. (C) The EIS analysis of the bare and each step of modified electrodes in [Fe(CN)_6_] ^–3/−4^ (0.2 mM). (D) Time profile of Aptamer/miRNA‐128 interaction based on CV techniques at different incubation time

Electrochemical techniques (CV, SWV, and EIS) were used to ensure the proper synthesis of the desired nanocomposite, as well as changes in the surface properties of the working electrode with different nanomaterial modifications. CV and SWV tests were conducted after each stage of working electrode modification for obtaining information about the surface changes due to the nanomaterials characteristics. As shown in Figure 5A and B, the highest current peak is related to the glassy carbon electrode without any modification, as it can exchange electrons with [Fe(CN)_6_]^–3/‐4^ without restriction. By stabilizing the RGO nanoparticles on the electrode, the electron exchange between the electrolyte and the electrode surface is mediated by a barrier containing oxygen atoms, making the redox reaction more limited. As a result, as shown in the red curves, the current peak decreases by 50.56%. Next, by adding Fe_3_O_4_ nanoparticles to the RGO sheets, the electron transfer is facilitated by the presence of Fe atoms, and as shown in the blue curves, the peak intensity of the current is 13.67% higher than before. Then, with the addition of AuNPs along with the residual sites on RGO sheets, the electron transfer is further increased by 0.95% (green curve) because of the high conductivity of gold atoms. AuNPs also provide a suitable substrate for binding aptamer molecules in the next step. Following incubation of aptamer on the nanocomposite, it increases the charge transfer resistance on the electrode surface and thereby decreases the current intensity by 0.72% (purple curve). Prior to the final step, the surface of the nanocomposite is coated with Nafion polymer. This polymer acts as a lattice that enables better decoration of aptamer molecules to bind to miRNA molecules. Nafion also prevents undesirable surface adsorption on the electrode surface, thereby enhancing biosensor accuracy and selectivity. As shown in the yellow curves, adding Nafion decreases the peak intensity of the current by 3.29% because some of the active sites are blocked. Finally, due to the hybridization of miRNA‐128 and aptamer, and consequently, surface deformation, the electron transfer rate and current peak intensity are significantly reduced by 15.70% according to the light blue curve. EIS is an electrochemical technique capable of providing useful information on charge transfer at the electrode interface. By modification of the electrode surface with various materials, the surface resistance of the electrode varied. In the Nyquist curve, the semicircular diameter represents the current transfer resistance (R_ct_). As expected from the results of the CV and the SWV in the EIS technique at each step, the semicircle diameter (Figure 5C) is proportional to the peak of the current intensity (Figure 5A,B). In any step that the current peak increases, the corresponding semicircle diameter is reduced in the Nyquist curve due to the raising of conductivity and surface area of the electrode.

After the modification process, the main parameter of miRNA‐128 concentration determination is the optimum time. The duration in which the electrode was incubated in a 2 mM [Fe(CN)_6_]^3‐/4–^ solution at a constant concentration of the analyte is displayed in Figure 5D. In order to investigate the response time of the biosensor, a constant concentration of miRNA‐128 loaded on the modified electrode surface, and after 5, 15, 25, 35, 45, and 55 min, respectively, CV technique conducted for obtaining the current peak difference. Then the experiment was performed in a 2 mM [Fe(CN)_6_]^–3/−4^ medium in the potential range of ‐0.50.7 V at a scan rate of 50 mV.s^–1^. The final result is presented in Figure 5D. As can be seen, the hybridization between the aptamer and miRNA‐128 is negligible at 5 min. The hybridization rate augmented with increasing time to 35 min, but it became stable in the 35–45 min period, indicating that the 35 min is the optimum response time for this biosensor.

### 3.2 miRNA‐128 detection in ferrocyanide electrolyte

**FIGURE 6:**
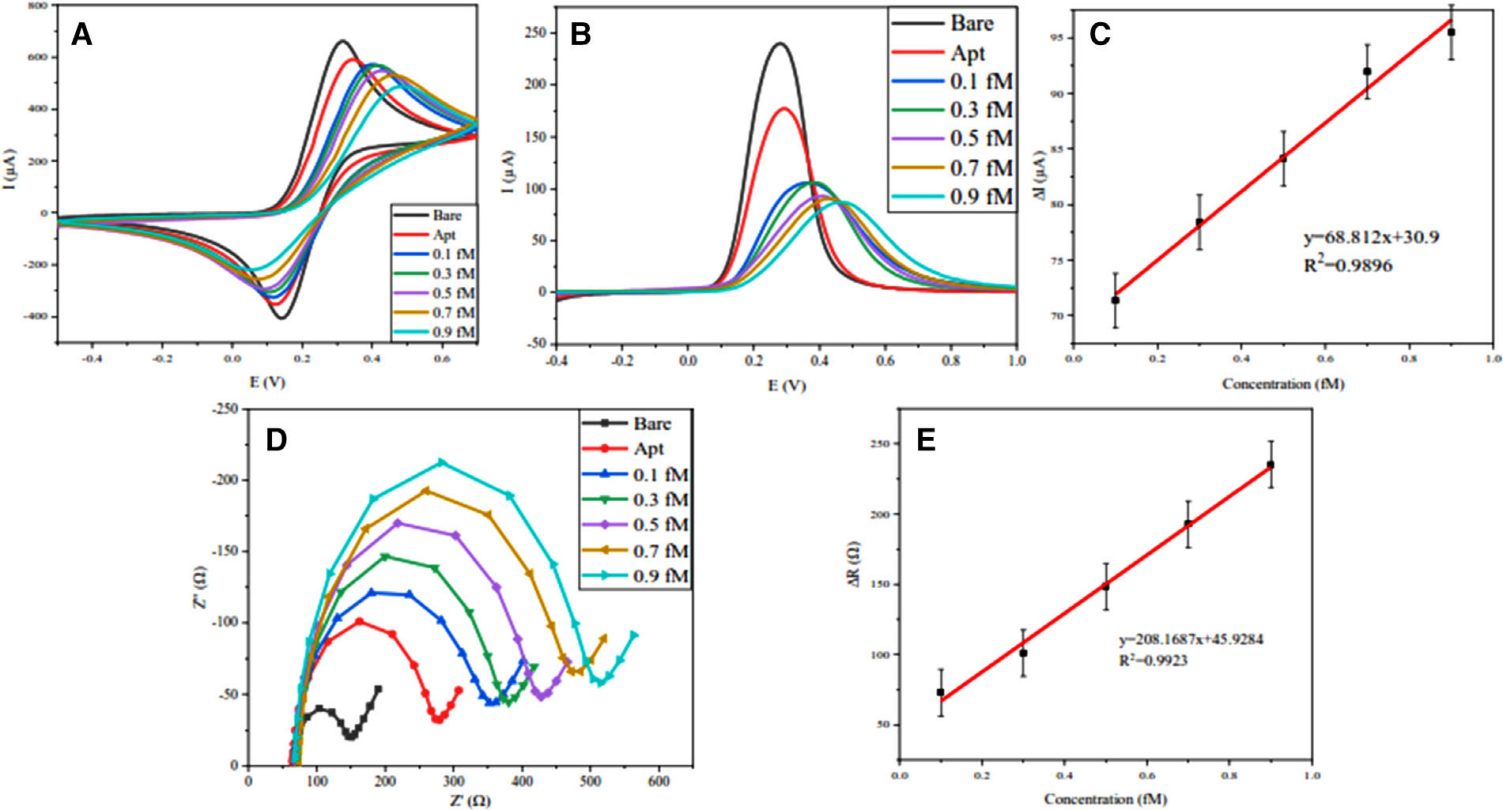
(A) CV voltammograms of Apt/Au/Fe_3_O_4_/RGO electrode [Fe(CN)_6_]^3−/4–^ media at 0, 0.1, 0.3, 0.5, 0.7, 0.9 fM of miRNA‐128. (B) SWV voltammograms of Apt/Au/Fe_3_O_4_/RGO electrode [Fe(CN)_6_]^3−/4–^ media at 0, 0.1, 0.3, 0.5, 0.7, 0.9 fM of miRNA‐128. (C) Calibration plot derived from SWV. (D) Nyquist diagrams of the electrode in [Fe(CN)_6_]^3−/4–^ media at 0, 0.1, 0.3, 0.5, 0.7, 0.9 fM of miRNA‐128. (E) Calibration plot derived from EIS

For concentration determination in [Fe(CN)_6_]^–3/−4^ media, first, the electrode base signal was obtained by CV, SWV, and EIS tests (black curves in Figure 6A,B,D). After that, the stabilization of the aptamer chain was confirmed in the same (red curves in Figure 6A,B,D). Concentrations from 0.1 fM to 0.9 fM was considered for this procedure. According to the results obtained in Figure 6A, B and D, at each step, as the concentration increased, the electron transfer resistance (Rct) increased. Therefore, the current intensity has decreased. As shown in Figure 6C and E, the calibration curves related to SWV and EIS tests are approximately linear. According to the formula limit of detection (LOD) = 3Sb.m^–1^, where Sb is the standard deviation for blank and m is the slope of the calibration graph, the LOD value for the present aptasensor is 0.0546 fM.

### 3.3 miRNA‐128 detection in PBS

**FIGURE 7:**
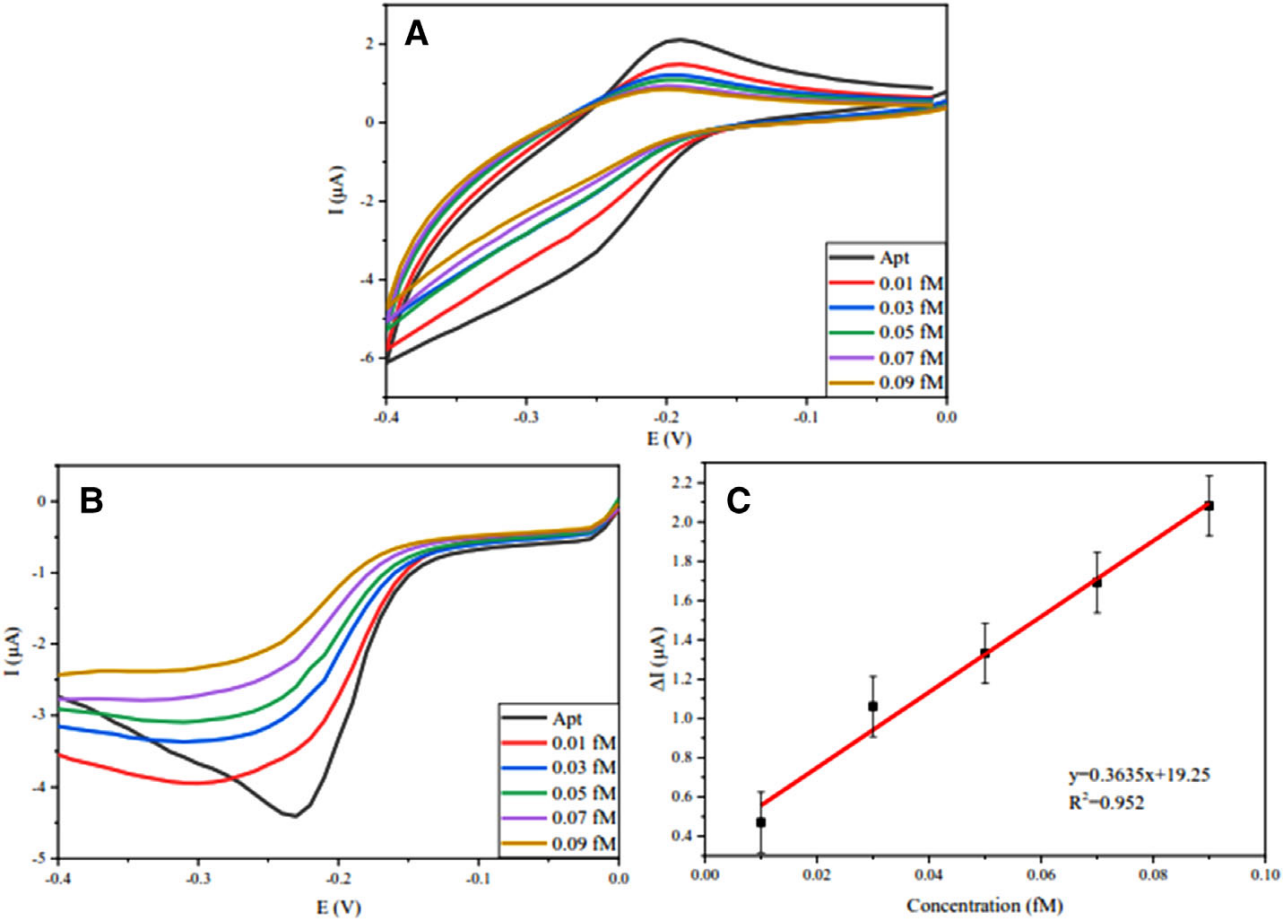
(A) CV voltammograms of labeled nanoprobe electrode in PBS media at 0, 0.01, 0.03, 0.05, 0.07 and 0.09 fM of miRNA‐128. (B) SWV voltammograms of labeled nanoprobe electrode in PBS media at 0, 0.01, 0.03, 0.05, 0.07 and 0.09 fM of miRNA‐128. (C) Calibration plot derived SWV

Despite the fact that potassium ferrocyanide produces a good electrochemical signal, it can be very hazardous. For this reason and also increasing the biosensor sensitivity, we used the nanoprobe electrode in PBS media with the application of methylene blue label as the electrochemical mediator. Other advantages of this procedure are the similarity of PBS media with human blood and reduction of electrochemical potential to 0. CV and SWV tests were used to investigate the changes in current intensity versus potential changes, the results of which can be seen in Figure 7A and B. As shown in these two figures, as the concentration of the analyte increases, the resistance of the electron transfer on the surface of the electrode increases due to the changes in surface configuration and blocking the active sites, resulting in a decrease in the peak current of the plot. It should be noted that the concentration range at this stage is from 0.01 fM to 0.09 fM, which is one‐tenth of the concentration range compared to the measurement in potassium ferrocyanide electrolyte. So it has much higher accuracy. The calibration curve for the SWV test is also plotted to determine the biosensor's performance in the predetermined range and calculate R^2^ in Figure 7C. The LOD is obtained by 0.005483, according to the formula mentioned above.

### 3.4 Repeatability

**FIGURE 8:**
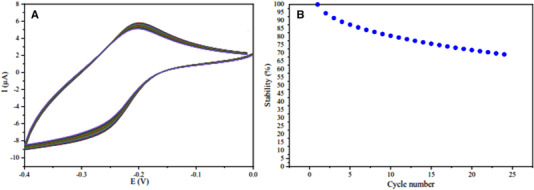
(A) CV voltammogram related to the stability test after 24 cycle. (B) Stability percentage after 24 cycles

One of the essential parameters related to biosensor reliability is its repeatability. So for the determination of repeatability, we measured the nanoprobe stability on the surface of the working electrode. To measure the stability, as shown in Figure 8A, after stabilizing the Apt/Au/Fe_3_O_4_/RGO probe on the electrode surface, the CV test was performed sequentially 24 times. The results of these 24 cycles show that there was no significant change in the voltammogram. Therefore, the stability of the biosensor is highly confirmed. Figure 8B also shows the points obtained from the same test, in which the percentage of probe stability on the electrode is determined in each cycle.

### 3.5 Diffusion control

**FIGURE 9:**
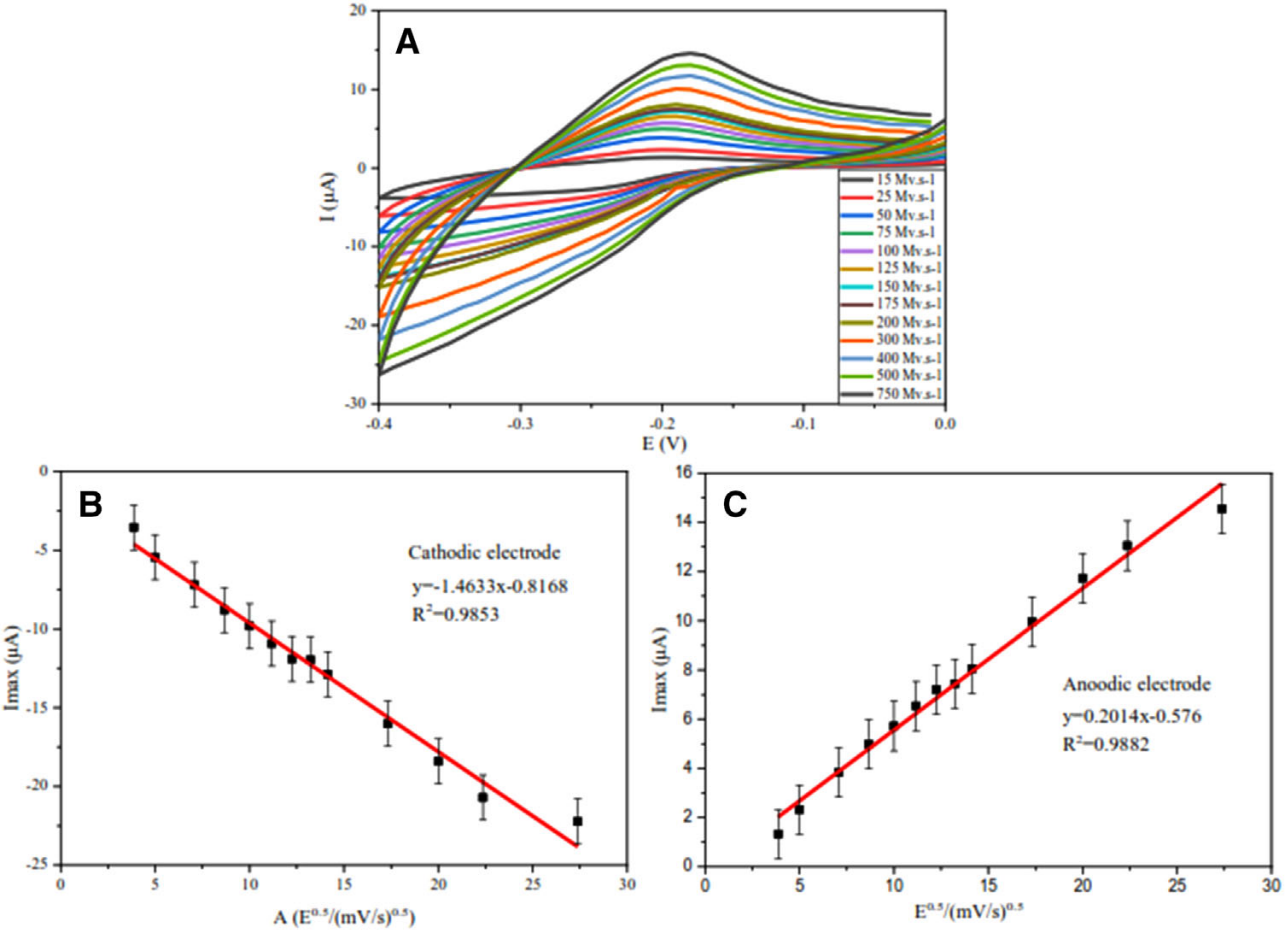
(A) CV voltammogram related to the diffusion control test. (B) Calibration plot of the return path of the diffusion control test. (C) Calibration plot of the forward path of the diffusion control test

Finally, the diffusion control test was performed to examine the relationship between the increase in scan rate and the maximum current obtained. Redox probe has good electrochemical performance if it has good diffusion control; which means, according to the Randles–Sevcik equation (at 25°C, $i_{p}=\;kn^{\frac{3}{2}}AD^{\frac{1}{2}}v^{\frac{1}{2}}C$, i_p_ = current maximum, *k* = 2.69×10^5^, *n* = number of electrons transferred in the redox event, *A* = electrode area in cm^2^, *D* = diffusion coefficient in cm^2^.s^–1^, *C* = concentration in mol.cm^–3^), as the scan rate increases, the maximum current will also increase. Moreover, by having the concentration of the species in the solution, the surface area of the working electrode, and the scan rate, the diffusion coefficient can be found with the help of the CV test [84, 85]. As shown in Figure 9A, as the scan rate increases, the thickness of the diffusion layer decreases, and as a result, the diffusion rate increases, resulting in an increase in the maximum electric current intensity. The test was performed with 13 different values for scan rates of 15, 25, 50, 75, 100, 125, 150, 150, 175, 200, 300, 400, 500, and 750 mV.s^–1^. Finally, in Figure 9B and C, the maximum current intensity calibration curves were plotted in two‐electrode modes (anode and cathode).

### 3.6 Selectivity

**FIGURE 10:**
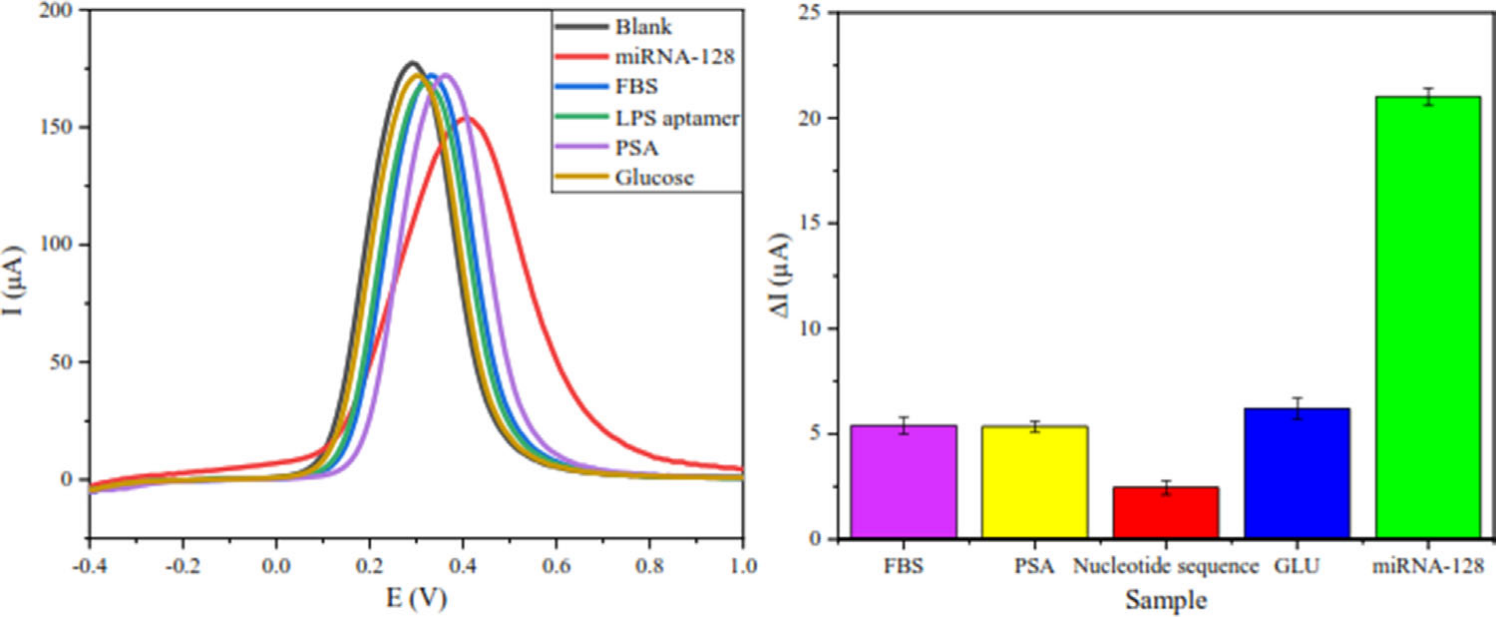
Comparsion of nanoprobe electrode signal response for miRNA‐128 (0.1 fM), LPS aptamer (100 μM), glucose (90 mg.dL^−1^), PSA (2.5 ng.mL^−1^), and BSA (5 ng.mL^−1^)

One of the key considerations for evaluating the output of a biosensor is its selectivity, which is the specificity of a sensor to a given analyte to prevent false results due to potentially interfering species. In addition to miRNA‐128, which is a desirable analyte, four other biomolecules with concentrations in the body of a healthy human were prepared to measure the selectivity of the present biosensor, and the biosensor response to them was recorded. As can be seen in Figure 10, the difference in current intensity due to miRNA‐128 stabilization on the working electrode is much higher than the other four materials (3.38 times glucose, 8.57 times the LPS aptamer (CTTCTGCCCGCCTCCTTCCTAGCCGGATCGCGCTGGCCAGATGATATAAAGGGTCAGCCCCCCAGGAGACGAGATAGGCGGACACTOD5 HPLC 5' Mod Amino Modifier ‐ NH2 C6), 3.92 times PSA and 3.88 times FBS), which confirms the high selectivity of the biosensor.

A comparison of prepared aptasensor with other reported electrochemical biosensors for detecting miRNAs is presented in Table 1. The results show the suitable performance of the prepared nanoprobe against other reports.

**TABLE 1:** Comparison of the analytical performance of the new nanoprobe with other electrochemical miRNA nanoprobes

miRNA name	Related illness	Linear range	LOD	Reference
miRNA‐155	Myocarditis	1 fM–100 pM	0.14 fM	[86]
miRNA‐21	Breast cancer	10 fM–1 nM	0.78 fM	[87]
miRNA‐21	Breast cancer	10 fM–10 μM	0.2 fM	[88]
miRNA‐182	Lung cancer	1 fM–0.1 nM	0.43 fM	[89]
miRNA‐128	Acute lymphoblastic leukemia	0.01 fM–0.09 fM	0.005483 fM	The present research

## 4 CONCLUDING REMARKS

The electrochemical nanoprobe electrode for the detection of miRNA‐128 was described for the first time in this research. The modified electrode displayed high selectivity to miRNA‐128, among other biomolecules, and had very low LOD in both label‐free and labeled techniques. Using methylene blue as a safer redox mediator caused the detection of miRNA‐128 at shallow potentials in PBS media with higher accuracy. Decoration of reduced graphene oxide sheets with Fe_3_O_4_NPs caused a suitable substrate for easier placement of gold nanoparticles on its surface, as well as increasing the conductivity of nanocomposite, which significantly helps to improve the performance and sensitivity of the sensor. Also, the binding of AuNPs on the reduced graphene oxide sheets provided a fast aptamer immobilization on the surface of the electrode and increased the electrochemical conductivity of reduced graphene oxide sheets. The stability test performed by the electrode marked by methylene blue showed that the system used after 25 tests is stable up to 69.19%. The results obtained from the diffusion control test also showed that the maximum current is directly related to the square of the scan. The results showed that the use of reduced graphene oxide‐magnetite‐gold nanoparticles significantly enhanced the detection limit and linearity range, which can be confirmed by comparing the results of previous research on the detection of other miRNAs. All in all, based on what has been achieved in current research, long‐lasting challenges in the field of cancer detection can be addressed by the commercialization of miniaturized aptamer‐based electrochemical biosensors, which are capable of detecting cancer biomarkers in very low concentrations in body fluids and even breath. This platform also can be integrated into wearable biosensors, which enables the real‐time monitoring of individuals.

## CONFLICT OF INTEREST

The authors have declared no conflicts of interest.
